# Analogical Assessment of Train-Induced Vibration and Radiated Noise in a Proposed Theater

**DOI:** 10.3390/s23010505

**Published:** 2023-01-02

**Authors:** Xiangming Liu, Yuchun Xiao, Huihuang Jiang, Yunlong Guo, Mengwen Yu, Wanzhong Tan

**Affiliations:** 1 China Academy of Railway Sciences Corporation Limited, Beijing 100081, China; 2 China Academy of Railway Sciences (Shenzhen) Research and Design Institute Co., Ltd., Shenzhen 518057, China; 3 Shenzhen Vibration and Noise Control Engineering Laboratory for Urban Rail Transit, Shenzhen 518057, China; 4 Bureau of Public Works of Shenzhen Municipality, Shenzhen 518042, China; 5 Department of Railway Engineering, Delft University of Technology, 2628CN Delft, The Netherlands

**Keywords:** train-induced vibration, radiated noise, analogical assessment, vibration acceleration level, A-weighted sound pressure, noise rating number, condition assessment

## Abstract

This study presents the analogical assessment of the train-induced vibration and radiated noise in a proposed theater. The theater is to be constructed in a region with crowded metro lines, and the assessment is implemented in an analogical building with comparable structural type and metro condition. Prior to the assessment, the comparability of the analogical building with the theater is validated using the train-induced ground vibration. With the same horizontal distance from the metro line, the train-induced vibration level in the analogical building is 9 dB higher than that in the construction site of the theater. Such results indicate that the lack of soil layers may lead to a dramatic increase in train-induced vibration in the building. In the staircase of the analogical building, the train-induced radiated noise reached 55 dB (A), which is 10 dB (A) higher than the daytime allowable level. As the most important indicator, the noise rating number in the cinema of the analogical building is *NR*-43, which put forward an enormous challenge on the construction of the theater with a denoise demand of 23 dB. The analogical method applied in this study provides an effective and practical way for the assessment of train-induced vibration and radiated noise in proposed vibration-sensitive buildings. The assessment results that provide necessary reference and support for the anti-vibration design will help guarantee the stage effect of the theater.

## 1. Introduction

Accompanied by the fast urbanization progress in China, the construction of urban rail transit, especially the metro, has developed rapidly as well in a lot of large cities. The gradually perfected metro network brings great convenience to the public travelling in the downtown area, while in the meantime leading to increasingly more vibration and noise problems. The excessive metro-train-induced vibration and noise not only affects the work and living conditions of nearby residents [1,2] but also results in a negative impact on historical buildings [3,4], sensitive equipment [5], etc. Therefore, prior to the construction of environmentally sensitive structures close to metro lines, the impact of train-induced vibration and radiated noise on the structure has to be effectively assessed in the preliminary design phase. In case the vibration or noise exceeds the allowable level, certain mitigation measures have to be implemented to guarantee the functionality of the structure.

Train-induced vibration and noise is a complex systematic problem, which can be affected by many factors including vibration sources, propagation paths and receivers. The vibration sources of the metro concern the whole vehicle–track–foundation system. It has been found that vehicle and track damping measures, such as dampers on the wheels [6] and rails [7], elastic clips [8], ladder slabs [9] and embedded [10] and floating slab tracks with damping pads [11,12,13,14] and steel springs [15], etc., can have a significant impact on the vibration levels and frequency characteristics. Besides the variation of track structures, the track condition is also an important influencing factor that cannot be ignored. The impact factors include but are not limited to the change of wheel and rail profiles due to wear and maintenance [16], the temperature force in the rail [17], different types of track irregularities [18] and installation quality of fastening clips [19], etc. Regarding the propagation path of the vibration, which is mainly the surrounding rock and soil of the railway foundation, the physical properties [20,21] as well as distribution of soil and rock layers [20,22] are considered as influential factors that may contribute to the attenuation of vibration. In the aspect of vibration and noise receivers, the foundation type [23], the height and weight of the structure [24], the position of measurement points [24,25,26] and the vibration isolation design [27,28], etc., can all influence the vibration and noise level.

To assess the impact of train-induced vibration and radiated noise on adjacent buildings, both experimental [29,30,31,32,33] and numerical [34,35,36,37,38] methods are commonly applied. Connolly et al. [29] analyzed many vibration records at several high-speed rail sites across Europe to obtain more insights into ground vibration prediction. Kouroussis et al. [30] measured and analyzed the ground vibration generated by different trains to obtain relevant parameters to model the train/track structure. Mouzakis et al. [31] proposed a method to determine the transfer function of vibration diffusion inside the given geological media based on field measurement and applied the transfer function to assess the train-induced vibration on nearby buildings. Sanayei et al. [32] analyzed the surface train- and subway-induced 3D vibrations in both inside buildings and open fields, respectively. Zou et al. [33] proposed predicted models for train-induced vibration and noise based on the existing model and validated it through field measurements in a metro depot. Ibrahim et al. [34] developed a track–soil–structure finite element model and analyzed the vibration mitigation effect of different trench techniques. López-Mendoza et al. [35] proposed a scoping model to predict train-induced vibration in buildings with the soil–structure interaction taken into consideration. Lopes et al. [36] proposed a 2.5D FEM-PML numerical approach for the prediction of vibrations induced in buildings due to railway traffic in a tunnel. Guo et al. [37] investigated the influence of some key parameters, e.g., train speed and fastener configurations, on the train-induced vibration acceleration level of a metro depot through numerical simulation. It can be seen that for numerical modelling and analysis, in situ measurements to some extent remain necessary for model validation [38]. In some cases, the numerical method is applied in combination with field measurement [39]. The entire process from vibration generation and propagation to noise radiation signifies that in situ measurement is still the most effective and intuitive method to assess train-induced vibration and noise.

It has to be noted that the impact of train-induced vibration and noise on proposed structures cannot be directly assessed through on-site measurement due to the lack of foundation and structure, while accurate numerical modelling and simulation is quite time-consuming and not efficient for engineering application. Considering that the assessed vibration and noise level will be used to guide the design of vibration mitigation measures, a slight deviation can be acceptable. Therefore, to assess the vibration and noise level of proposed structures, analogical measurement and analysis based on an existing building with comparable structural type and relative location to the metro is a practical option.

Recently in Shenzhen, a top-level theater close to several metro lines was proposed. To guarantee the stage effect of the theater, the train-induced vibration and noise in the theater have to be kept within the stipulated limits. Therefore, the goal of this study is to assess the expected levels of the train-induced vibration and radiated noise in the theater. To achieve this goal, measurements and analysis based on an analogical building with similar relative location to the metro and structural type as the theater are implemented. To ensure the applicability of the analogical assessment, the analogical building is validated using the metro train-induced ground vibration responses. The train-induced vibration and radiated noise in both the public areas and the cinema in the analogical building are measured, and the results are analyzed using the indicators with the consideration of frequency weighting. The outcomes of the analogical assessment will be further applied as key references to provide necessary guidance for the anti-vibration design of the theater.

## 2. Overview of the Theater

The theater is proposed to be constructed in one of the urban core districts in Shenzhen, China. Containing a dream theater with more than 1000 seats and a multi-functional performing space with more than 500 seats, it is positioned as a milestone project to enhance the image of Shenzhen as an international metropolis.

**Figure 1:**
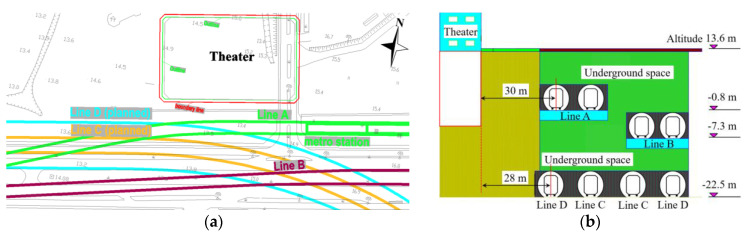
The plane (**a**) and section (**b**) positional relationships of the theater and the metro lines.

The urban core region of Shenzhen has the highest metro density in China with over 1 km/km^2^ in double lines, and the train-induced vibration and noise problems due to the operation of metro lines are particularly prominent. In the construction site of the theater, there are currently two metro lines (Line A and Line B) passing on the south. In the near future, there will be another two metro lines (Line C and Line D) also passing on the south. The plane and section positional relationships of the theater and the metro lines are shown in Figure 1.

The shortest horizontal distance between the theater (designed outline) and Metro Line A (middle line of the closed tunnel) is 30 m. To the south of Line A, Line B passes over with the shortest horizontal distance from the theater of around 80 m. The proposed Metro Line C and Line D will pass to the south of the theater with the shortest distance of 28 m, and these two lines are currently in the stage of preliminary design. Based on the principle of preconception, priorities are given to Line A and Line B in the assessment of train-induced vibration and radiated noise. In return, the assessment results can also help provide a reference for the anti-vibration design of Metro Line C and Line D.

## 3. Assessment Methods

The assessment of the train-induced vibration and radiated noise is aimed to be applied to determine the vibration mitigation requirement for the theater. Therefore, the assessment work is performed based on the Chinese standards for building construction. Specifically, the impact of train-induced vibration and radiated noise in the public area of the theater are assessed according to standard JGJ/T 170-2009 [40], and the noise level in the theater and the performing space of the theater are assessed according to standard GB/T 50356-2005 [41].

According to the above standards, the indicator for the vibration assessment is the maximum vibration level in 1/3 octave band frequency divisions (in the range of 4–200 Hz, expressed by *VL*_max_). The indicator for the radiated noise assessment is the equivalent continuous A-weighted sound pressure (in the frequency range of 16–200 Hz, expressed by *L*_Aeq_). In addition, the noise level in the dream theater and the performing space is the noise rating (*NR*) value in the frequency range of 31.5–8000 Hz. All the indicators are briefly explained in this section.

### 3.1. Experimental Tools

The key components for the assessment of the train-induced vibration and radiated noise are the accelerometer for the tunnel wall and ground vibration measurements and the sound level meter for the radiated noise measurements. The main configurations of these sensors are listed in Table 1.

### 3.2. Vibration Assessment Method

The vibration acceleration level can be calculated through Formula (1).

(1) $$VL=20\mathrm{lg}\frac{a}{a_{0}}$$

where *a* is the root mean square acceleration value, and *a*_0_ is the reference acceleration value. For train-induced vibration, *a*_0_ = 10^−6^ m/s^2^. For discrete data, the root mean square acceleration value can be calculated through Formula (2):

(2) $$a=\sqrt{\frac{1}{n}(\sum_{j=1}^{n}a_{j}^{2})}$$

where *n* is the data length of the measured discrete acceleration signal. The *VL*_max_ can be calculated through Formula (3):

(3) $$VL_{\max}=\underset{k=1\to n}{\max}(VL_{k}+w_{k})$$

where *VL_k_* is the vibration acceleration level in each 1/3 octave frequency band, and *w_k_* is the corresponding weighting factor. The recommended values of *w_k_* in 4–200 Hz are provided in the international standard ISO 2631-1: 1997 [42]. The *VL*_max_ corrects to an integer by rounding.

### 3.3. Radiated Noise Assessment Methods

The impact of train-induced radiated noise in the theater is assessed in two dimensions. One dimension is for the public area of the theater except the dream theater and the multi-functional performing space, the assessment method is the *L*_Aeq_; the other dimension is for the dream theater and the multi-functional performance space. The assessment method is the noise rating (*NR*) value. The details of radiated noise assessment methods are presented below.

#### 3.3.1. Equivalent Continuous A-Weighted Sound Pressure

The A-weighted Sound pressure (*L*_A_) can be calculated through Formula (4):

(4) $$L_{A}=20\mathrm{lg}\frac{p}{p_{0}}$$

where *p* is the root mean square sound pressure, and *p*_0_ is the reference sound pressure. For air-born sound, *p*_0_ = 2 × 10^5^ Pa. The sound pressure A-weighting factors in 1/3 octave frequency bands are provided in the international standard IEC 61672-1:2013 [43].

The mean energy value of A-weighted sound pressure in the specified measurement time is *L*_Aeq_, which can be calculated through Formula (5).

(5) $$L_{\mathrm{Aeq}}=10\mathrm{lg}\frac{1}{n}\sum_{i=1}^{n}10^{0.1L_{\mathrm{AE},\;i}}$$

where *n* is the number of train passages, and *L*_AE_, *i* is the A-weighted sound pressure of train *i*. It has to be noted that the measured radiated noise is effective only when it is more than 3 dB (A) higher than the background noise. In case the difference between measured radiated noise and background noise is 3–9 dB (A), the measurement results have to be amended by 1–3 dB (A) [40].

#### 3.3.2. Noise Rating Number

The noise rating (*NR*) curve for the assessment of noise levels inside various types of buildings was proposed in the international standard ISO/R 1996:1971 [44]. In GB/T 50356-2005, the *NR* curve is applied to the acoustic control for theater, cinema and multi-use auditorium. In this method, the sound pressure levels in octave-band frequencies of 31.5–8000 Hz are measured, and the sound pressure limit in each frequency band can be calculated through Formula (6).

(6) $$L_{\mathrm{PB}}=A+B\times NR$$

where $L_{\mathrm{PB}}$ is the allowable sound pressure level in each frequency band, A and B are constant values that correspond to each frequency band [41], and *NR* is the sound pressure level in the frequency range of 1000 Hz.

Using this method, the *NR* number is determined by the tangent point of the sound pressure curve. Specifically, we plot the fitted sound pressure curve on the *NR* curve map, and the *NR* curve with the highest tangent point is defined as the *NR* number of the measured sound pressure.

### 3.4. Allowable Vibration and Noise Levels

Combining the requirements of the theater with the stipulations of the standards, the allowable *VL*_max_ value is 62 dB, and the allowable *L*_Aeq_ value is 42 dB (A).

The noise control values in the dream theater and the multi-functional performing space are both *NR*-20. The sound pressure levels in the corresponding octave frequencies are listed in Table 2.

## 4. Measurements and Analysis in the Theater Construction Site

In order to investigate the impact of both Line A and Line B, the vibration measurements at the construction site of the theater consist of two parts: the vibration of the excitation source measured on the tunnel wall of the metro lines and the vibration along the propagation path between the theater and the metro lines measured on the ground. Both parts are presented in this section.

### 4.1. Metro Information

The main configurations of Metro Line A and Line B in the region close to the construction site of the theater (Figure 1) are given in Table 3. Both lines use the same type of metro train, while the designed train velocity of Line B is much higher than that of Line A. The track in both lines is the same ordinary monolithic track bed without additional damping. Another key difference between these two lines is the tunnel construction method. The tunnels of Line A were constructed using the cut and cover method, while for Line B, the tunnels were constructed using the shield method.

### 4.2. In-Track Vibration Measurements and Analysis

**Figure 2:**
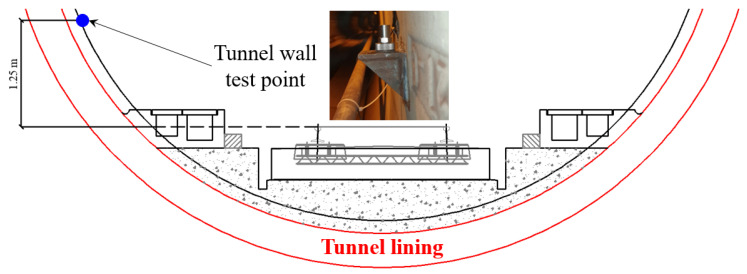
Setup of tunnel wall vibration test point.

The measurement of the in-track vibration indicates the vibration intensity of the excitation source. According to the technical guidelines for environmental impact assessment—urban rail transit (HJ 453-2018 [45])—to assess the excitation source vibration intensity, the recommended test position for underground metro lines is on the tunnel wall with 1.25 m above the rail surface, as shown in Figure 2.

**Figure 3:**
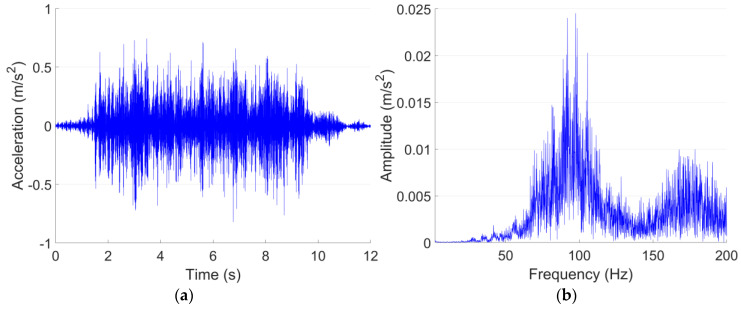
Typical tunnel wall acceleration responses in time (**a**) and frequency (**b**) domain in Line A.

**Figure 4:**
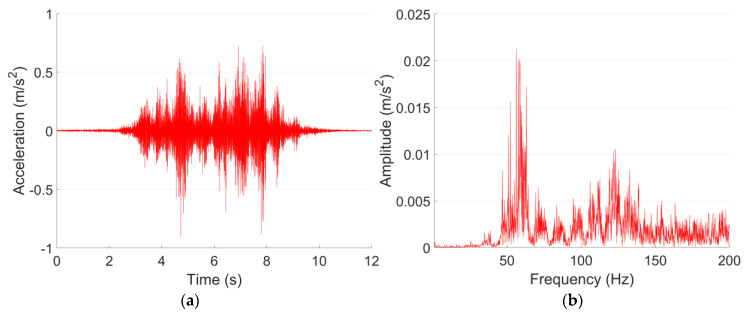
Typical tunnel wall acceleration responses in time (**a**) and frequency (**b**) domain in Line B.

The vibration of both Line A and Line B are measured on the tunnel wall. According to JGJ/T 170-2009 [40], to calculate the root mean square value of *VL*_max_, at least five trains within the same velocity range need to be recorded. In each vibration test point in this study, the mean value of *VL*_max_ is calculated based on 20 pass-by trains, and the acceleration responses are lowpass filtered with a cut-off frequency of 200 Hz. The representative acceleration responses of both lines in the time and frequency domain are shown in Figure 3 and Figure 4.

It can be seen from Figure 3a and Figure 4a that despite the differences in train velocity and train length, the tunnel wall acceleration responses of Metro Line A and Line B in 0–200 Hz are at the same level, with the maximum values of 0.8–0.9 m/s^2^. For Line A, the vibration responses are rather stable. While for Line B, the vibration responses fluctuated,, which indicate different deterioration levels of the passing wheels. The dominant frequencies of Line A and Line B are 98 Hz and 56 Hz, respectively. Such a difference in the frequency domain responses is possibly caused by the structural type of the tunnel (Table 3). 

**Figure 5:**
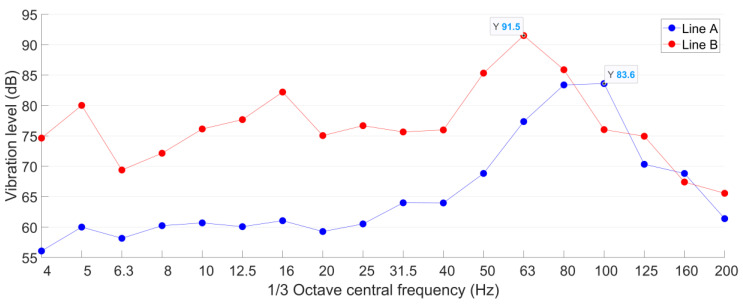
Tunnel wall 1/3 octave frequency division vibration acceleration level of Line A and Line B.

The 1/3 octave frequency division vibration acceleration levels of both lines on the tunnel wall are calculated, as presented in Figure 5.

It can be seen from Figure 5 that the vibration acceleration level of Line B is in general higher than that of Line A. The *VL*_max_ of Line B is 92 dB, which is 8 dB higher than that of Line A. The 1/3 octave frequency bands corresponding to the *VL*_max_ are 100 Hz in Line A and 63 Hz in Line B, which are consistent with the dominant frequencies of both lines (Figure 3b and Figure 4b). Such results indicate the high correlation between these two expression forms of vibration.

In the frequency bands from 4 Hz to 63 Hz, the vibration acceleration levels in Line B are more than 10 dB higher than those of Line A. It has to be noted that low frequency vibration responses usually propagate farther in the soil than high frequency vibration responses. From this point of view, although Line A is closer to the proposed theater, the vibration induced by Line B with higher *VL*_max_ and lower dominant frequency may propagate farther than that of Line A. Therefore, to figure out the metro line that has the dominant impact on the proposed theater, tunnel wall vibration responses are not enough. The ground vibration in the construction site of the theater has to be further measured and analyzed.

### 4.3. Ground Vibration Tests and Analysis

**Figure 6:**
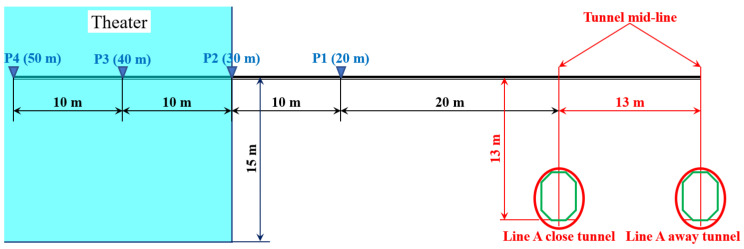
Ground vibration setup in the construction site of the theater.

The ground vibration test points are set-up along the direction perpendicular to the metro lines, as demonstrated in Figure 6. The horizontal distances of the test points P1–P4 from the mid-line of the Line A tunnel are 20–50 m with intervals of 10 m.

**Figure 7:**
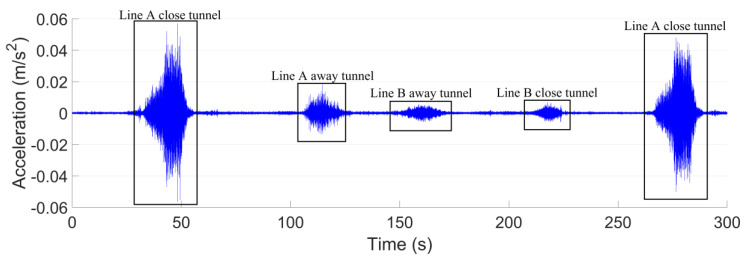
Ground vibration responses measured in test point P1.

The ground vibration measurements are performed with continuous sampling, and the responses corresponding to trains are recognized by the train records in the metro stations. A short period of typical time-domain ground vibration responses obtained from test point P1 (Figure 6) is shown in Figure 7. The corresponded tunnels of the vibration sources are marked.

**Figure 8:**
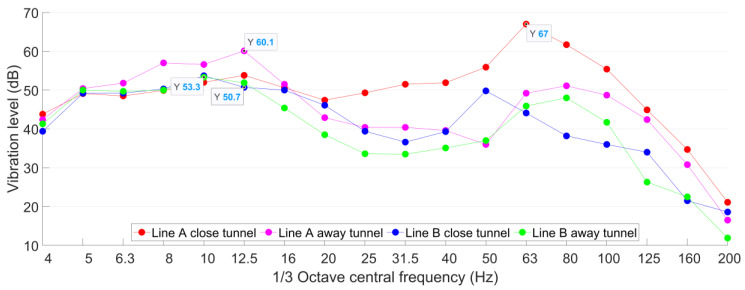
Ground 1/3 octave frequency division vibration acceleration level of Line A and Line B in P1.

It can be seen from Figure 7 that the ground vibration responses caused by the trains from the Line A close tunnel are much higher than the others. For both the close Line A tunnel and the further away tunnel of Line B, the train-induced ground vibrations are at the same level. The ground vibration level of Line A and Line B in the test point P1 is shown in Figure 8.

The *VL*_max_ induced by the train from the Line A close tunnel is 67 dB with the corresponded frequency band of 63 Hz. Such a result is 7 dB higher than that of Line A away tunnel, and more than 13 dB higher than those of Line B tunnels. It has to be noted that in the frequency range of 6.3–16 Hz, the vibration responses due to the trains from the Line A away tunnel are higher than those from the Line A close tunnel, which is likely caused by the interference of the metro tunnel (Line A close tunnel) on the vibration propagation.

**Figure 9:**
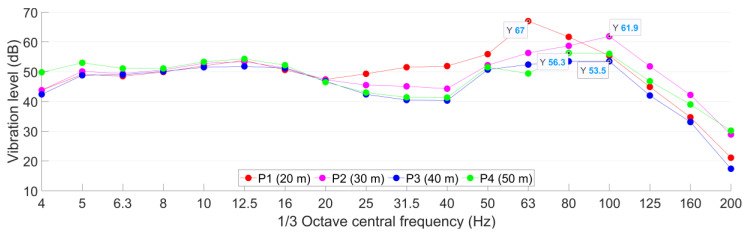
Ground 1/3 octave frequency division vibration acceleration level of the Line A close tunnel in different test points P1–P4.

It can be concluded that the ground vibration induced by the trains from the Line A close tunnel has the highest impact on the proposed theater. Therefore, in the further analysis of vibration propagation, only the trains from the Line A close tunnel are taken into account. The vibration levels from P1 to P4 induced by the trains from the Line A close tunnel are calculated, as presented in Figure 9.

It can be seen from Figure 9 that the *VL*_max_ attenuated from 67 dB in the test point P1 to 62 dB in P2 and further decreased to 54 dB and 56 dB in P3 and P4, respectively. In the frequency range of 4–20 Hz, the vibration levels are relatively stable and did not decrease with the increase of metro distance. In the test point P4, the vibration levels in 4–20 Hz are even higher than those in P1–P3. In terms of an existing building, the vibration acceleration level of P2 represents the impact of train-induced ground vibration on the building [45]. Considering that the metro-train-induced ground vibration has no noticeable fluctuation between daytime and nighttime; the vibration acceleration level of P2 is already equal to the nighttime limit (62 dB, Section 3.4).

The impact of train-induced vibration in adjacent buildings is affected by many factors, not only from the track [7,8,9,10,11,12,13,14,15,16,17,18,19] but also from the geological condition [20,21,22,23] and the structure itself [27,31,32]. Therefore, the ground vibration measurement results are not enough to assess the expected vibration acceleration level in the theater. In addition, the radiated noise level cannot be assessed without the structure. To have a better assessment of the metro-train-induced vibration and radiated noise in the theater, the analogical measurement and analysis in an existing building with a similar geological footprint and structural type as the theater are implemented, as presented in the section below.

## 5. Measurements and Analysis in the Analogical Building

In this section, the vibration and noise assessment based on an analogical building is presented. Prior to that, the selection of the analogical building is briefly introduced, and the applicability validation of the analogical building using the ground vibration is performed and analyzed.

### 5.1. Analogical Building Selection

**Figure 10:**
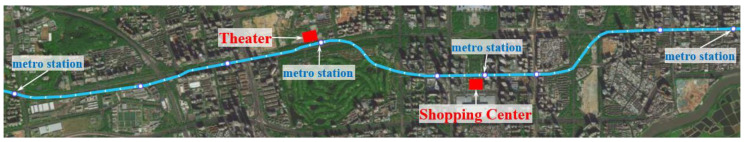
Route map of Metro Line A.

Due to the variation of the construction period, standard and track damping requirement, Metro Line A is to some extent different from all the other lines in terms of tunnel construction method, buried depth, geological conditions, etc. Therefore, the analogical building is to be selected along Line A where the geological condition, tunnel depth and track structure are almost identical. The route map of Line A is shown in Figure 10.

After comparing all the buildings along Line A, a shopping center (Figure 10) with the same minimum horizontal distance from Line A, similar floor space and foundation depth with the theater is selected as the analogical building for the assessment of train-induced vibration and noise. Additionally, there is a cinema in the shopping center located close to the side of Line A, which can further apply to help assess the expected radiated noise level in the dream theater and the multi-functional performing space in the theater. The main train, track, tunnel and geological conditions of both buildings are compared in Table 4. It can be seen that despite slight differences in metro tunnel depth and structure foundation depth, the shopping center has very high comparability with the theater.

### 5.2. Ground Vibration Validation

**Figure 11:**
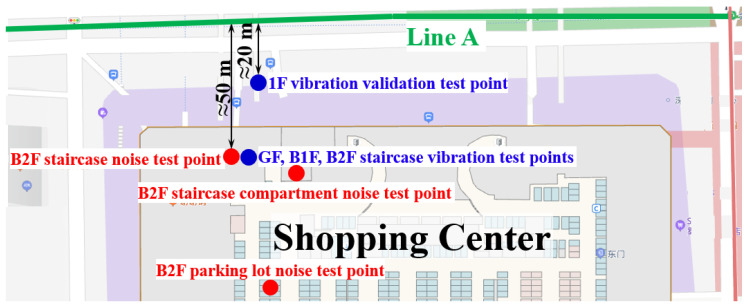
Test points set up in the public area of the shopping center.

**Figure 12:**
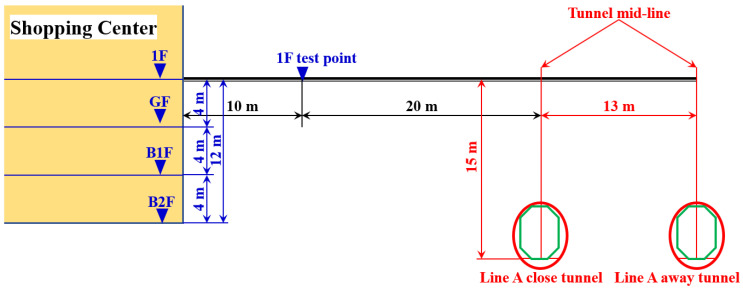
Cross-section diagram of mutual position relationship between the shopping center and Metro Line A.

To ensure the reliability of analogical assessment results, the train-induced vibration in the shopping center is validated using the ground vibration results from the construction site of the theater. The vibration validation test point is setup on the first floor (1F) out of the shopping center, and the horizontal distance from the Line A close tunnel is around 20 m, as shown in Figure 11. The mutual position relationship between the shopping center and Metro Line A is shown in Figure 12.

**Figure 13:**
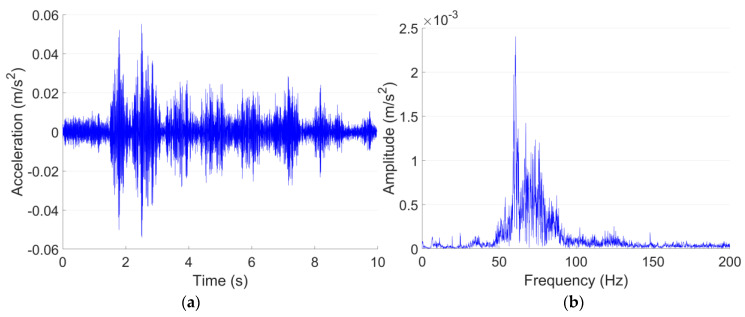
Typical acceleration responses in time (**a**) and frequency (**b**) domain in 1F test point in the shopping center.

**Figure 14:**
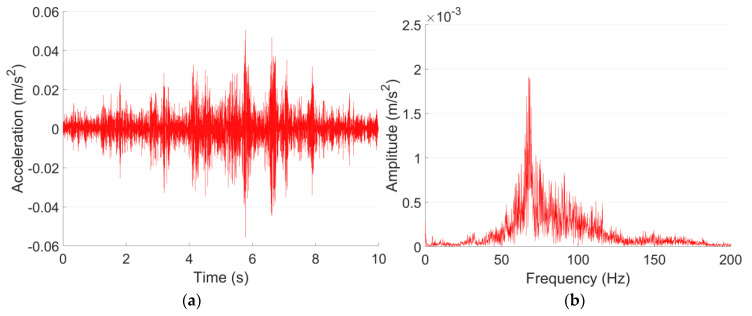
Typical acceleration responses in time (**a**) and frequency (**b**) domain in P1 in the site of the theater.

The ground vibration of the shopping center is validated using the vibration measurement results of test point P1 in the site of the theater, which is also 20 m from the Line A tunnel in the horizontal direction. The representative acceleration responses of both test points in the time and frequency domain are shown in Figure 13 and Figure 14. Similar to the tunnel wall vibration responses, the ground vibration responses are lowpass filtered with the cut-off frequency of 200 Hz.

**Figure 15:**
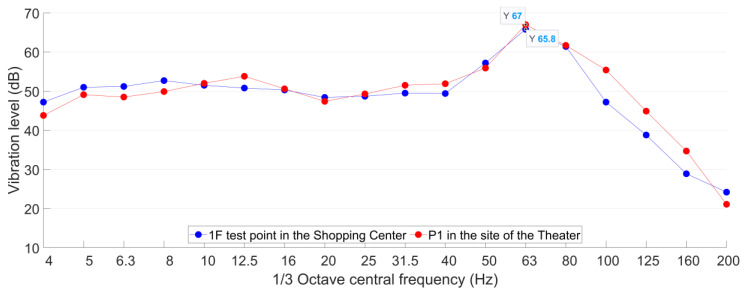
Comparison of 1/3 octave frequency division vibration acceleration level in 1F test point in the shopping center and P1 in the site of the theater.

Figure 13 and Figure 14 indicate that the Line A metro train-induced ground vibration responses in both test points in 1F in the shopping center and P1 in the site of the theater are quite close to each other. The maximum accelerations in both test points are around 0.05 m/s^2^, and the dominant frequencies are both around 60 Hz. The comparison of the 1/3 octave frequency division vibration acceleration level of both test points is given in Figure 15.

It is indicated in Figure 15 that the *VL*_max_ in the 1F test point in the shopping center and P1 in the site of the theater are 66 dB and 67 dB, respectively. The corresponding 1/3 octave frequency bands are both 63 Hz. Despite slight discrepancies in the absolute values in each frequency band, the 1/3 octave frequency division vibration acceleration level in both test points is highly consistent. Such results further prove that the metro condition in the shopping center is quite close to that in the theater, and it can be applied as the analogical building to help assess the expected metro-train-induced vibration and noise in the theater.

### 5.3. Analogical Measurements and Analysis

To comprehensively assess the train-induced vibration and noise, the analogical measurements and analysis are implemented in two parts:

- The vibration and noise measurements and analysis in the public area such as the staircase, staircase compartment and parking lot to simulate the public area of the theater;
- The vibration and noise measurements and analysis in the cinema simulate the dream theater and the multi-functional performing space in the theater.

Both parts are presented in the section below.

#### 5.3.1. Public Area Measurements and Analysis

**Figure 16:**
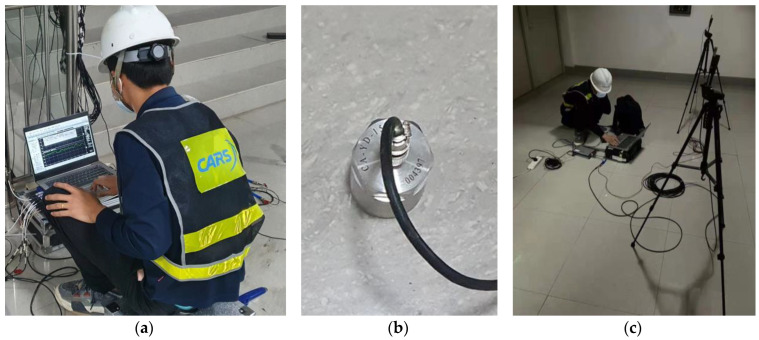
Accelerometer (**a**,**b**) setup in the staircase and sound level meters (**c**) setup in the compartment.

The measurements in the public area consist of three vibration test points and three noise test points. The positions of these test points are demonstrated in Figure 11, and the photos of the scene of the accelerometer and sound level meter setup are shown in Figure 16.

**Figure 17:**
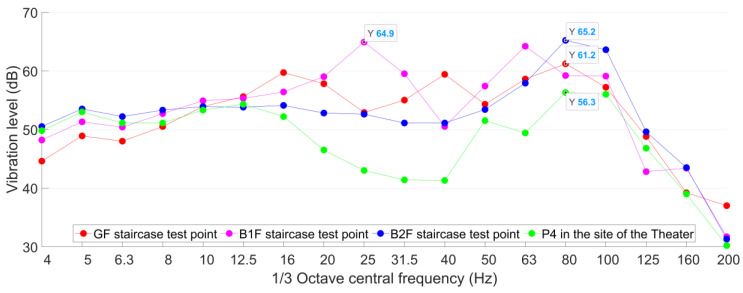
The1/3 octave frequency division vibration acceleration levels of the staircase test points in the shopping center compared with P4 in the site of the theater.

The ground vibration test points in the public area are, respectively, set up in the staircase on the ground floor (GF), the basement to the first floor (B1F) and the basement to the second floor (B2F), as shown in Figure 11. The horizontal distances of the test points from the Line A tunnel are all around 50 m (Figure 11). The measured vibration acceleration levels of these test points are calculated and presented in Figure 17.

It can be seen from Figure 17 that in the test point in B1F, *VL*_max_ = 65 dB with the corresponding 1/3 octave central frequency band of 25 Hz. In the test point in B2F, *VL*_max_ = 65 dB with the corresponding 1/3 octave central frequency band of 80 Hz. In both B1F and B2F test points, the maximum acceleration level exceeded the allowable value for 3 dB. For the test point in GF, *VL*_max_ = 61 dB, which is just within the allowable level. Although the staircase vibration test points have the same horizontal distance from the Line A tunnel, the measured vibration responses showed different *VL*_max_ values as well as the corresponded frequency bands (80 Hz in GF and B2F test points, 25 Hz in B1F test points). In the low-frequency bands of 4–8 Hz, the vibration responses in high stairs are lower than those in low stairs. Such differences can be explained by the different straight-line distances from the Line A tunnel (Figure 12).

The results of test point P4 on the site of the theater are distributed in Figure 17 as well. It can be seen that the vibration responses in the staircase are much higher than that in test point P4 in the construction site of the theater. Such results indicate that the train-induced vibration can propagate father in the structure due to the lack of damping soil. It can be concluded that the expected train-induced vibration in the public area will be 5–9 dB higher than the current ground vibration.

**Figure 18:**
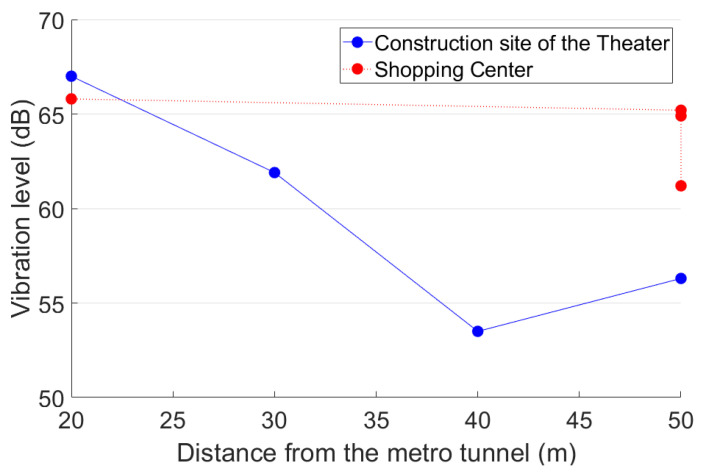
Development of ground vibration along the horizontal distance from the metro tunnel.

The developments of the vibration level with the horizontal distance from the metro tunnel in both the construction site of the theater and the shopping center are presented in Figure 18. In the construction site of the theater, the vibration level dramatically decreased with the increase of the horizontal distance from the metro tunnel. In the shopping center, the attenuation is rather limited due to the lack of damping soil.

It is shown in the vibration measurement results that the worst vibration situation appears in B2F. Therefore, the noise test points in the public area are set-up in B2F. Specifically, one test point in the staircase compartment (fully enclosed space, dimension = 8 × 12 × 4 m^3^, 60 m from Line A), one test point in the staircase (half enclosed space, dimension = 7 × 13 × 2 m^3^, 50 m from Line A) and one test point in the parking lot (open space, height = 4 m, 110 m from Line A), as shown in Figure 11.

**Figure 19:**
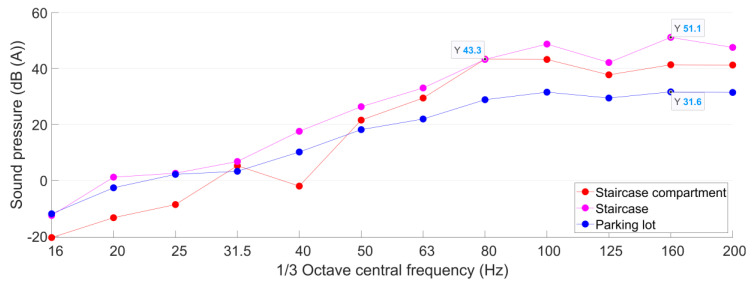
A-weighted sound pressure curves in the shopping center B2F test points.

The train-induced radiated noise is measured continuously in these three test points with more than 10 passing trains recorded, and the typical A-weighted sound pressure curves are shown in Figure 19.

Using Formula (5), the mean values of the equivalent continuous A-weighted sound pressure (*L*_Aeq_) in the B2F noise test points are calculated based on the responses of 10 continuously pass-by trains. Then, the results of *L*_Aeq_ are amended based on the sound pressure of the background noise obtained in each test point. The amended results of *L*_Aeq_ are given in Table 5.

Similar to the metro-train-induced vibration, the radiated noise has no distinct variation from day to night. The measured responses can be compared with both daytime and nighttime limits. Figure 19 and Table 5 show that the B2F staircase has the highest train-induced radiated noise. The amended *L*_Aeq_ = 55 dB (A), which is 13 dB (A) higher than the allowable value. In the staircase compartment, the amended *L*_Aeq_ is lower than that in the staircase, but still exceeds the allowable value for 7 dB (A). In the parking lot noise test point, which has an open space and is 110 m from the metro tunnel, the *L*_Aeq_ is only 4 dB (A) lower than the allowable value. Such results indicate the poor acoustic environment in the shopping center.

#### 5.3.2. Cinema Measurements and Analysis

**Figure 20:**
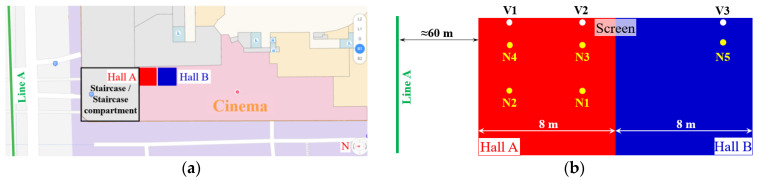
Schematic diagram of the relative location between cinema halls and metro Line A (**a**) and the test points set up (**b**).

The cinema in the shopping center is constructed across B1F and B2F. The vibration and noise measurements in the cinema are implemented in Hall A and Hall B. Hall A is located next to the staircase for the public area vibration and noise measurement, and Hall B is located next to Hall A. The plane position relationship between the cinema and Line A is shown in Figure 20.

**Figure 21:**
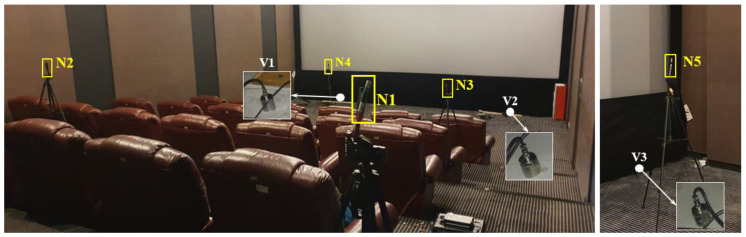
Setup of sound pressure test points in Hall A (**left**) and Hall B (**right**) in the cinema.

In the measurements in the cinema, two vibration test points (V1 and V2) and four noise test points (N1–N4) are set up in Hall A, one vibration test point (V3) and one noise test point (N5) are set up in Hall B, as demonstrated in Figure 20b and Figure 21. The shortest horizontal distance from Hall A to the metro tunnel is around 60 m, and the width of each hall is 8 m. To avoid the influence of the carpet on the vibration measurement, the accelerometers are set up under the screen in B2F. To better analyze the train-induced radiated noise in the cinema hall, all the main acoustic sources including the air conditioner and broadcast are turned off during the measurements.

**Figure 22:**
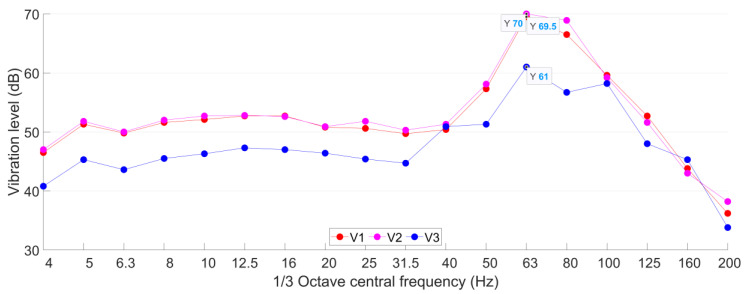
The 1/3 octave frequency division vibration acceleration levels in the cinema.

The vibration acceleration levels obtained from the test points in the cinema are presented in Figure 22. It can be seen that the responses of the two vibration test points in Hall A are consistent with each other. The *VL*_max_ is 70 dB and the corresponded frequency band is 63 Hz. Since both test points V1 and V2 are set up on the same beam, the measured vibration responses to some extent represent the vibration of the beam. It has to be noted that although the test points in Hall A in the cinema are more than 10 m farther from the Line A tunnel than those in the staircase, the *VL*_max_ in Hall A is more than 5 dB higher than that in the B2F staircase test point and 8 dB higher than the allowable level in the theater. Such results indicate that the cinema hall with a larger space is not good for the attenuation of train-induced vibration.

In the test point V3 in Hall B, the *VL*_max_ is 9 dB lower than those in Hall A with the same corresponded frequency band (63 Hz). The vibration level in Hall B is lower than that in Hall A in almost all the frequency bands. Such results indicate that the wall between the two halls effectively reduced the propagation of ground vibration.

**Figure 23:**
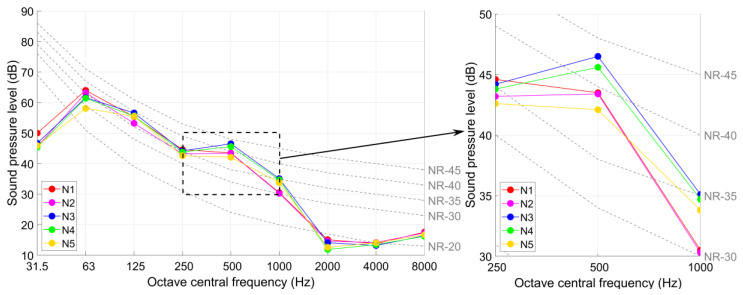
*NR* curves in the noise test points in the cinema.

The train-induced radiated noise as well as the background noise in the test points N1–N5 in the cinema are measured simultaneously with the ground vibration. The *NR* curves of the radiated noise are presented in Figure 23. Because all the acoustic sources in the cinema are turned off, the measured background noise levels are all below 20 dB, which are even out of the measurement range of the sound level meter (Table 1). Therefore, the influence of background noise does not need to be taken into account.

The measurement results of the radiated noise (*L*_Aeq_ and *NR* values) in the test points N1–N5 are given in Table 6.

It can be seen from Figure 22 and Table 6 that the maximum *NR* value obtained from noise measurements in the cinema halls is *NR*-43 (in test point N3) with corresponding octave central frequency band of 500 Hz. Compared with the allowable value of *NR*-20 value in the theater, the noise reduction requirement in the theater is more than 23 dB. 

It can be noticed that the radiated noise in the cinema is presented in three levels, which are *NR*-42–*NR*-43 in test points N3 and N4, *NR*-40 in test points N1 and N2, and *NR*-38 in test point N5, respectively. Such results indicate that in a room with the layout of stadium seating (e.g., cinema hall, theater room, etc.), the train-induced radiated noise level in the rear seats can be slightly lower than that in the front seats. Compared to the noise level in test points N3 and N5, the noise attenuation effect of the wall between two halls is around 4 dB.

It can also be seen from Table 5 and Table 6 that the results of *L*_Aeq_ in the cinema halls (40–42 dB (A)) are much lower than those in the staircase (55 dB (A)) and the staircase compartment (49 dB (A)). Such results indicate that the larger room space with sound absorption material (wall decoration of the cinema halls) can help reduce the radiated noise level in the room.

## 6. Conclusions and Discussion

This paper presented the assessment of the metro-train-induced vibration and radiated noise on the proposed theater through analogical measurements and analysis. The analogical building was selected based on the comparability with the theater and validated using the train-induced ground vibration responses. Based on the vibration and noise results in both the public area and the cinema in the analogical building, the following conclusions can be drawn.

- The train-induced vibration presents higher responses in the analogical building. With the same horizontal distance (50 m) from the metro tunnel, the ground vibration in the staircase of the analogical building was up to 65 dB, which is 9 dB higher than that in the construction site of the theater. In the cinema of the analogical building (60 m from the metro tunnel), the train-induced ground vibration was up to 70 dB, which is already 5 dB higher than the daytime allowable level for the theater. Such results indicate that the expected vibration level in the theater will be much higher than now measured at the construction site, which can be explained by the excavation of soil that reduced the soil damping effect.
- The highest equivalent continuous A sound pressure obtained in the public area of the analogical building was 55 dB (A), which is 10 dB (A) higher than the allowable level of the theater. The noise rating number in the cinema of the analogical building was up to *NR*-43. Compared with the allowable level of *NR*-20 for the dream theater and multi-functional performing space, the noise reduction requirement for the theater is more than 23 dB, which puts forward a challenging task for the theater anti-vibration design and construction.
- The expected high impact of train-induced vibration and radiated noise on the proposed theater is not only due to the mutual positional relationship between the theater and the metro tunnel, but also due to the lack of anti-vibration design of the current metro line. Such a fact puts forward requirements on the planned metro lines. To guarantee the stage effect of the theater, vibration mitigation measures have to be fully considered.

This study is a new attempt at the assessment of the train-induced vibration and radiated noise on a proposed vibration-sensitive building. Due to the lack of structure, the ground vibration obtained from the construction site is not enough to demonstrate the expected impact of trains. In this case, the analogical method that can quickly and relatively accurately assess the expected vibration and radiated noise levels is an efficient and practical way for engineering application.

## Figures and Tables

**Table 1:** Configurations of the applied sensors.

Sensor Type	Test Object	Measuring Range	Resolution	Frequency Range
Accelerometer	Tunnel wall and ground acceleration	±7 m/s^2^	0.00025 m/s^2^	0.1–500 Hz
Sound level meter	Radiated noise	20–146 dB	0.01 dB	10–20,000 Hz

**Table 2:** The sound pressure levels of the *NR*-20 curve in the corresponding octave frequency bands.

**Octave Frequency (Hz)**	31.5	63	125	250	500	1000	2000	4000	8000
**Sound Pressure Level (dB (A))**	69	51	39	31	24	20	17	14	13

**Table 3:** Major configurations of Line A and Line B.

Items\Lines	Line A	Line B
Train type/Axle load	Metro Type A/17 tones	
Marshalling number	6	8
Train length	140 m	184 m
Passing velocity	60 km/h	110 km/h
Tunnel construction method	Cut and cover	Shield
Track/Rail type	Ordinary monolithic track bed/CN 60	
Depth of rail top	15 m	21 m
Shortest distance from the theater	30 m	80 m

**Table 4:** Track and geological conditions of the theater and the shopping center.

Items	Theater	Shopping Center
Metro track type	The ordinary integral track bed	
Train velocity	60 km/h	
The horizontal distance from Line A	≈30 m	
Tunnel rail surface depth	13 m	15 m
Structure foundation depth	15 m	12 m
Geological condition	Plain fill, grit, gravelly clay, completely decomposed granite	
Composition at the bottom of tunnels	Gravelly clay	

**Table 5:** Amended results of *L*_Aeq_ in the B2F noise test points (dB (A)).

B2F Noise Test Point	Radiated Noise	Background Noise	Difference	Amendment	Amended Noise
Staircase	55	37	18	0	55
Staircase compartment	49	30	19	0	49
Parking lot	39	32	7	1	38

**Table 6:** The measurement results of the radiated noise in the test points N1–N5.

Test Points	N1	N2	N3	N4	N5
*L*_Aeq_ (dB (A))	42	40	42	41	40
*NR* value	*NR*-40	*NR*-40	*NR*-43	*NR*-42	*NR*-38
